# Pain Competency Evaluation in Dementia (PACED) Scale – Validity and Reliability

**DOI:** 10.1093/geroni/igaf122.1336

**Published:** 2025-12-31

**Authors:** Yo-Jen Liao, Ying-Ling Jao, Marie Boltz, Nai-Ching Chi, Diane Berish, Terrence Murphy

**Affiliations:** The Pennsylvania State University, University Park, Pennsylvania, United States; The Pennsylvania State University, University Park, Pennsylvania, United States; The Pennsylvania State University, University Park, Pennsylvania, United States; University of Iowa, Iowa City, Iowa, United States; The Pennsylvania State University, University Park, Pennsylvania, United States; The Pennsylvania State University, University Park, Pennsylvania, United States

## Abstract

People with dementia experience undermanaged pain and often rely on nurses for pain management. Therefore, it is essential to understand nurses’ pain management competency in dementia. Existing pain competency scales are mostly subjective and lack focus on people with dementia. This study describes the development and establishment of validity and reliability of the Pain Competency Evaluation in Dementia (PACED) scale. PACED is an observational scale developed from a review of relevant literature, protocols, and guidelines. It includes 24 items in 5 categories. Each item is rated from 0=not performed to 3=accurate. Total score is calculated as History Takingx15% +Documentationx15% +Pain Assessmentx30% +Physical and Functional Assessmentx20% +Pain Treatmentx20%. Content validity was established with pain and/or dementia experts using the Delphi survey approach. We calculated Item-level Content Validity Index (I-CVI; range = 0-1, higher is better) focused on relevance and clarity. Undergraduate nursing students and Registered Nurses completed two virtual case scenarios in nursing home (NH) setting. Two raters were trained to observe and rate nurses’ pain management competency using the PACED scale to establish inter-rater and intra-rater reliability. Seven pain and dementia experts completed two rounds of Delphi Survey, and PACED was reduced to 16 items with relevance I-CVI=1.0 and clarity I-CVI=0.99. The raters were trained until interclass correlation (ICC)>0.7 at least 3 times. The raters demonstrated excellent inter-rater (ICC=0.971) and intra-rater (ICC=0.958) reliability for PACED. Next, the internal consistency of PACED will be examined. PACED will be used to evaluate nursing home nurses’ pain management competency in dementia.

